# Heterogeneity of Rheumatoid Arthritis–Associated Interstitial Lung Disease by Longitudinal Forced Vital Capacity Trajectory and Associations With Disease Outcomes

**DOI:** 10.1002/acr.25620

**Published:** 2025-11-13

**Authors:** Bryant R. England, Yangyuna Yang, Punyasha Roul, Tate M. Johnson, Scott M. Matson, Grant W. Cannon, Brian C. Sauer, Jeffrey R. Curtis, Joshua F. Baker, Ted R. Mikuls

**Affiliations:** ^1^ VA Nebraska‐Western Iowa Health Care System and University of Nebraska Medical Center Omaha; ^2^ Kansas University Medical Center Kansas City; ^3^ Salt Lake City VA Health Care System and University of Utah Salt Lake City; ^4^ University of Alabama at Birmingham; ^5^ Corporal Michael J. Crescenz VA Medical Center and University of Pennsylvania Philadelphia

## Abstract

**Objective:**

We aimed to identify unique disease trajectories within rheumatoid arthritis–associated interstitial lung disease (RA‐ILD) based on longitudinal forced vital capacity (FVC) values and their associated clinical outcomes.

**Methods:**

We performed a cohort study of RA‐ILD within the Veterans Health Administration from 1999 to 2021. Patients with RA‐ILD were identified with validated algorithms. Group‐based trajectory modeling was used to identify unique groups based on longitudinal FVC values in a primary cohort with baseline and follow‐up FVC values and an overall cohort with at least one FVC value. Associations of group assignment with survival and respiratory hospitalization were tested in multivariable Cox regression models adjusting for initial FVC.

**Results:**

We derived three FVC trajectory groups in the primary cohort (n = 1,092) and eight in the overall cohort (n = 5,172). A total of 82.5% (primary cohort) and 54% (overall cohort) of patients with RA‐ILD belonged to progressive groups with FVC percent predicted change ranging from −2.16 to −10.78 (primary) and −0.73 to −1.56 (overall) per year. Relative to the stable group, slow (adjusted hazard ratio [aHR] 1.55 and 1.38) and rapid (aHR 2.07 and 1.77) progressors in the primary cohort had an increased risk of death and respiratory hospitalization, respectively. In the overall cohort, patients with a progressive FVC trajectory also experienced an increased risk of death (aHR range 1.18–2.69) and respiratory hospitalization (aHR range 1.29–2.78).

**Conclusion:**

Unique disease trajectories based on longitudinal FVC values are independently associated with the risk of death and respiratory‐related hospitalization in RA‐ILD. Recognition of these unique RA‐ILD trajectories could inform management and monitoring.

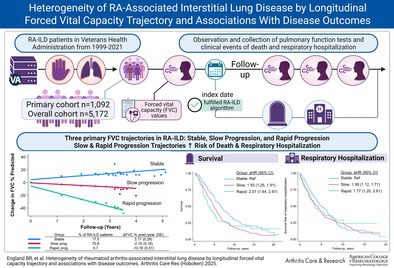

## INTRODUCTION

Interstitial lung disease (ILD) is an inflammatory and fibrotic diffuse lung disease that affects between 10% and 40% of people with rheumatoid arthritis (RA).^1^ Individuals afflicted with RA‐ILD suffer from reduced quality of life, poor survival, and a high degree of health care use.^2^, ^3^, ^4^ Recognizing the challenge providers face managing RA‐ILD in the absence of high quality evidence, the American College of Rheumatology (ACR) and American College of Chest Physicians (CHEST) recently developed the first clinical practice guidelines for the treatment of systemic autoimmune and rheumatic disease–associated ILD, including RA‐ILD.^5^ In that guideline, all treatment recommendations for RA‐ILD were conditional in nature because of the limited evidence and the phenotypic variability in RA‐ILD among patients. This substantial heterogeneity occurring in patients with RA‐ILD complicates disease management.

**SIGNIFICANCE & INNOVATIONS**

- Rheumatoid arthritis–associated interstitial lung disease (RA‐ILD) is a heterogeneous disorder with recognized variability by ILD pattern, change in pulmonary function (eg, forced vital capacity [FVC]), and disease outcomes.
- Using group‐based trajectory modeling of a large RA‐ILD cohort, we identified slowly progressive, rapidly progressive, and stable FVC disease trajectory groups, with the majority of patients with RA‐ILD belonging to progressive FVC decline groups.
- Patients characterized by slow or rapid FVC progression experienced an increased risk of death and respiratory‐related hospitalization, despite the rate of FVC decline being below thresholds used for eligibility criteria in antifibrotic clinical trials.
- Our findings suggest that reliance on the progression thresholds used as part of therapeutic regulatory trials in ILD may be overly stringent to inform real‐world treatment decisions in RA‐ILD.

RA‐ILD is commonly categorized according to the historadiologic ILD pattern. Usual interstitial pneumonia (UIP) is the most common pattern in RA‐ILD, with nonspecific interstitial pneumonia being the most common non‐UIP ILD pattern.^6^ Although genetic risk factors (eg, *MUC5B* promoter variant) differ by ILD pattern, and patients with RA‐ILD with a UIP pattern appear to have a higher risk of death than those with other patterns,^7^, ^8^, ^9^ ILD pattern was not considered a factor influencing treatment recommendations in the ACR/CHEST guidelines.^5^ Supporting this, an observational study recently demonstrated the ability of azathioprine, mycophenolate mofetil, and rituximab to all stabilize forced vital capacity (FVC), findings that did not differ by ILD pattern.^10^ Similarly, post hoc analyses of the INBUILD trial found similar slowing of FVC decline with nintedanib compared with placebo among patients with progressive fibrosing ILD, regardless of ILD pattern.^11^ Thus, other indicators of disease heterogeneity besides ILD pattern on chest computed tomography (CT) are needed to guide RA‐ILD management.

Although the management implications of ILD pattern in RA‐ILD remain to be fully elucidated, the importance of considering disease progression by pulmonary function test (PFT) parameters is becoming more apparent. In fact, based on the aforementioned INBUILD trial,^11^ nintedanib received United States Food and Drug Administration approval for ILD specifically with a progressive fibrotic phenotype. Recognizing the importance of characterizing longitudinal pulmonary function in RA‐ILD, recent studies have preliminarily explored whether there are underlying differences in the clinical disease course within RA‐ILD.^12^, ^13^ Two studies identified trajectories corresponding to improvement and/or stability, slow progression, and rapid progression that are predictive of disease outcomes (eg, death), though these reports were limited by small sample sizes (n = 140–172 RA‐ILD patients) and single‐center study populations.^12^, ^13^ Thus, our understanding of the range of different RA‐ILD disease trajectories that occur in large, real‐world RA‐ILD populations remains poorly understood.

The purpose of this study was to identify unique trajectories of FVC change over time among a national‐scale, real‐world population of patients with RA‐ILD within the Veterans Health Administration (VA), the largest integrated health care system in the United States. Subsequently, we aimed to evaluate the prognosis of patients within these unique RA‐ILD trajectories. We hypothesized that there would be distinct trajectories of FVC among patients with RA‐ILD that corresponded to differential prognosis.

## PATIENTS AND METHODS

### Design and population

We performed a cohort study of patients with RA‐ILD in the VA between 1999 and 2021. Patients with RA‐ILD were identified based on fulfilling validated algorithms for RA and ILD. For RA, we required the presence of two or more RA diagnostic codes, rheumatologist diagnosis of RA, and receipt of a disease‐modifying antirheumatic drug (DMARD) or positive autoantibody (rheumatoid factor [RF] or anti–cyclic citrullinated peptide [anti‐CCP] antibody).^14^, ^15^, ^16^ For ILD, we required the presence of two or more ILD diagnostic codes and either a pulmonologist diagnosis of ILD, completion of chest CT and PFTs, or completion of lung biopsy. Individuals with diagnostic codes for other connective tissue diseases (eg, systemic lupus erythematosus, inflammatory myositis, systemic sclerosis) or pneumoconioses were excluded. Such algorithms have >90% positive predictive value (PPV) for RA and >80% to 85% PPV for RA‐ILD.^17^, ^18^ The study index date was the date patients fulfilled the RA‐ILD algorithm (ie, latest of required components), which was at least two years after their first VA health care encounter for 88.2% of patients. For our primary RA‐ILD cohort, we required an FVC value within three years before the index date and at least one FVC value within five years after the index date. Because the disease course often impacts FVC testing in real‐world settings, which could introduce selection bias, we also created a broader overall RA‐ILD cohort that only required patients to have at least one FVC value during the study period. This study received institutional review board (IRB) approval (VA central IRB #1619487 and University of Utah IRB #12917). Patients and the public were not involved in this study.

### FVC values

In the primary cohort, FVC values were evaluated within three years before the index date to five years after the index date. In the overall cohort, all available FVC values were included. FVC values were obtained from two sources. First, when PFT equipment was compatible with discrete data storage, FVC values were obtained from the VA Corporate Data Warehouse (CDW). Second, recognizing frequent PFT equipment incompatibility with the VA CDW, we extracted additional FVC values from electronic health record notes using a validated natural language processing (NLP) tool.^19^ All FVC values were analyzed in this study as FVC percent predicted values.

### Survival and respiratory hospitalization

In cohort analyses evaluating the association of unique disease trajectories with disease outcomes, the study outcomes were all‐cause mortality and respiratory‐related hospitalization. Vital status was obtained through linkage with the National Death Index, a highly valid approach for ascertaining vital status in veterans.^20^ Respiratory‐related hospitalizations were obtained from the VA CDW, non‐VA care billed to the VA (eg, Fee Basis), and linked Centers for Medicare and Medicaid Services (CMS) data. Hospitalizations were considered respiratory related if the primary discharge diagnosis had respiratory‐related International Classification of Diseases, Ninth Revision, (ICD‐9; 466.x–519.x) or ICD‐10 (J09.x–J99.x) diagnostic codes. These events were assessed after the index date.

### Clinical variables

Additional clinical data were obtained for descriptive purposes and to serve as covariables in regression models. These included demographics, smoking status, body mass index (BMI), comorbidity burden, RA autoantibody status (RF and anti‐CCP antibody), elevated erythrocyte sedimentation rate or C‐reactive protein, DMARD use, and antifibrotic use (nintedanib and pirfenidone). These variables were obtained from the VA CDW before or on the index date. Comorbidity burden was assessed using a count of up to 43 chronic conditions, each requiring multiple diagnostic codes on separate days before the index date, as in prior work.^21^ Medication use was evaluated before the index date. We categorized patients into calendar year periods based on the year fulfilling the RA‐ILD algorithm (1999–2005, 2006–2011, 2012–2021).

### Statistical analysis

Group‐based trajectory modeling was used to define unique clusters of patients based on longitudinal FVC trajectory. Group‐based trajectory modeling is a finite mixture statistical modeling approach to approximate the unknown distribution of trajectories within a population.^22^ These approaches have been previously used in RA‐ILD and systemic sclerosis–associated ILD.^12^, ^13^, ^23^ Group‐based trajectory modeling was implemented using the “traj” Stata plugin (StataCorp). Consistent with best practices for group‐based trajectory modeling,^22^ the selection of optimal numbers of groups was informed by model fit characteristics (eg, change in Akaike information criterion) and the size of the smallest group combined with clinical interpretability. Linear models were used because polynomial functions were tested and did not improve model fit (data not shown). In the primary RA‐ILD cohort, change in FVC from the initial value was modeled. In the broader overall RA‐ILD cohort, the actual FVC value was modeled, producing both an intercept (ie, initial FVC value) and slope (ie, change in FVC values). For individuals in the overall cohort with only one FVC value, the proximity of this value to the estimated trajectories determined group assignment.

Patient characteristics were descriptively summarized overall and by group assignment. Associations of FVC trajectory group assignment with survival and respiratory‐related hospitalization were assessed using unadjusted Kaplan–Meier curves and in separate multivariable Cox regression models. Covariates in these models were age, sex, race, smoking status, BMI category, calendar year period, comorbidity burden, and baseline FVC. The proportional hazards assumption was tested by Schoenfeld residuals and negative log‐log survival plots. Schoenfeld residuals were significant, which was identified to be related to missing or unknown race data, a covariate in our regression models. No other Schoenfeld residuals were significant and log‐log survival plots did not indicate violation. Therefore, Cox models were considered appropriate for our analyses. Missing covariables were addressed using the missing indicator method. All analyses were completed using Stata version 18 within the VA Informatics and Computing Infrastructure.

## RESULTS

### Study population

We studied 5,172 patients with RA‐ILD who had 18,007 available FVC values in the overall RA‐ILD cohort, of which 1,092 patients with RA‐ILD (with 4,038 FVC values) fulfilled additional eligibility criteria for the primary cohort. Patients in both cohorts were predominantly male, had a mean age of 68 years, and had a frequent‐smoking history (Table 1). Comorbidity burden was high with a mean of 11.4 (SD 5.3) comorbidities and 49.7% having chronic obstructive pulmonary disease (COPD). Prednisone (67.8% and 72.4%) and conventional synthetic DMARDs (csDMARDs; 64.8 and 69.1%) were frequently used in the primary and overall cohorts, whereas biologic/targeted‐synthetic DMARDs were used less frequently (28.7 and 32.9%). Baseline antifibrotic use in the RA‐ILD population was rare during this study period (0.2%).

**Table 1:** Patient characteristics of study cohorts*

	Primary RA‐ILD cohort (n = 1,092)	Overall RA‐ILD cohort (n = 5,172)
Age, y	68.0 (9.5)	68.8 (9.9)
Male	1,012 (92.7%)	4,776 (92.3%)
Race		
Asian/multiple races	24 (2.2%)	122 (2.4%)
Black	162 (14.8%)	708 (13.7%)
White	782 (71.6%)	3,882 (75.1%)
Missing	124 (11.4%)	460 (8.9%)
Calendar year		
1999–2005	284 (26.0%)	943 (18.2%)
2006–2011	256 (23.4%)	1,382 (26.7%)
2012–2021	552 (50.5%)	2,847 (55.0%)
Smoking status		
Never	133 (12.2%)	606 (11.7%)
Former	346 (31.7%)	1,619 (31.3%)
Current	463 (42.4%)	2,331 (45.1%)
Missing	150 (13.7%)	616 (11.9%)
Comorbidity burden	10.1 (4.8)	11.4 (5.3)
COPD	481 (44.0%)	2,569 (49.7%)
RF/CCP antibody status		
Seronegative	190 (17.4%)	903 (17.5%)
Seropositive	747 (68.4%)	3,479 (67.3%)
Missing	155 (14.2%)	790 (15.3%)
ESR/CRP		
Normal	230 (21.1%)	1,143 (22.1%)
High	696 (63.7%)	3,402 (65.8%)
Missing	166 (15.2%)	627 (12.1%)
Prednisone^a^	740 (67.8%)	3,744 (72.4%)
csDMARDs^a^	708 (64.8%)	3,573 (69.1%)
b/tsDMARDs^a^	313 (28.7%)	1,702 (32.9%)
Antifibrotic use^a^	2 (0.2%)	9 (0.2%)

* Values are represented as mean (SD) or n (%). b/tsDMARD, biologic/targeted‐synthetic disease‐modifying antirheumatic drug; CCP, cyclic citrullinated peptide; COPD, chronic obstructive pulmonary disease; CRP, C‐reactive protein; csDMARD, conventional synthetic disease‐modifying antirheumatic drug; ESR, erythrocyte sedimentation rate; RA‐ILD, rheumatoid arthritis–associated interstitial lung disease; RF, rheumatoid factor.

^a^ Use before the study index date.

### FVC trajectories

#### Primary RA‐ILD cohort

**Figure 1:**
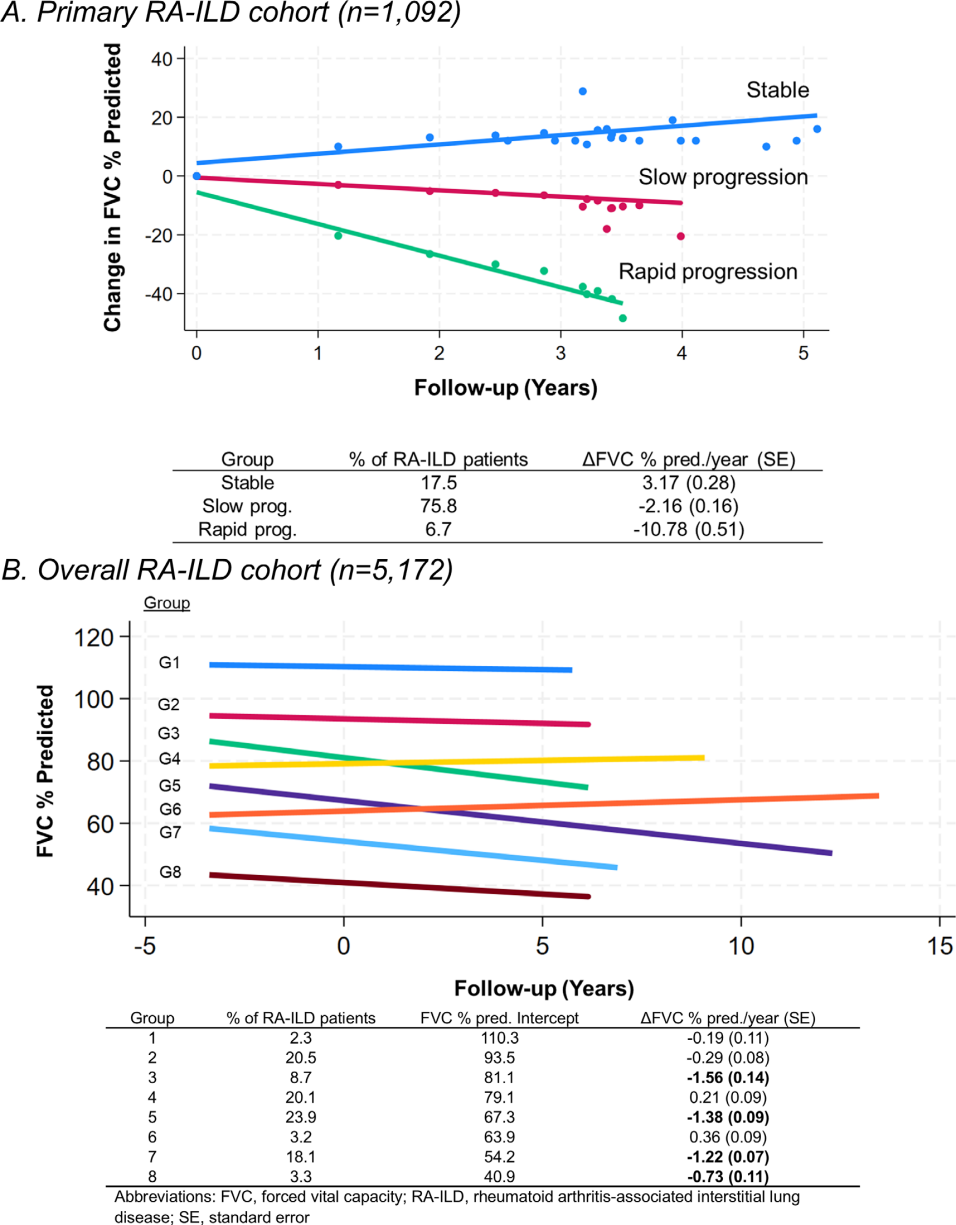
Unique trajectories of FVC measurements among patients with RA‐ILD. FVC percent predicted over time in patients with RA‐ILD in the Veterans Affairs Health Care System for (A) the primary RA‐ILD cohort and (B) the overall RA‐ILD cohort. Clusters of patients with RA‐ILD defined by unique FVC trajectories using group‐based trajectory modeling. The dots represent the mean values at each time point. The percentage of patients belonging to each group, initial FVC percent predicted (overall RA‐ILD cohort only), and change in FVC percent predicted over time are summarized in the tables. FVC, forced vital capacity; G, group; RA‐ILD, rheumatoid arthritis–associated interstitial lung disease.

We identified three FVC trajectory groups (Supplemental Table 1) that were labeled as stable, slow progression, and rapid progression based on change in FVC over time (Figure 1). Only 17.5% of patients with RA‐ILD had a stable FVC trajectory. Most patients were categorized into the slow progression group (75.8%), with a mean FVC change of −2.16% per year. Rapid progression occurred among 6.7%, with a mean FVC change of −10.78% per year. Those with progressive trajectories were older, more likely men and former smokers, and less likely to have comorbid COPD (Supplemental Table 2).

#### Overall RA‐ILD cohort

In the overall cohort, eight unique FVC trajectory groups were identified (Supplemental Table 3) that varied both by the initial FVC value (ie, intercept) and rate of FVC change during follow‐up (Figure 1). Of the eight RA‐ILD trajectory groups, we categorized four groups as progressive based on their observed rate of change. The mean decline in FVC percent predicted per year ranged from −0.73% to −1.56% in the progressive group. A total of 54% of the patients with RA‐ILD belonged to one of the four progressive RA‐ILD trajectory groups. The intercept for initial FVC percent predicted also varied among these progressive ILD patients, ranging from 40.9% to 81.1%. Among the 46% of patients with RA‐ILD belonging to the four nonprogressive groups, mean change in FVC percent predicted per year ranged from –0.29% to 0.36%. In these groups, the intercept for initial FVC ranged from 63.9% to 110.3%.

Those with a progressive FVC trajectory were more likely to have been followed from earlier calendar year periods (Supplemental Table 4). Patient characteristics by all eight unique FVC trajectory group assignments are provided in Supplemental Table 5. Differences among these trajectory groups were observed for age, calendar year period, smoking status, RF/anti‐CCP seropositivity, and csDMARD use.

### Prognosis in primary RA‐ILD cohort by trajectory

#### Mortality

**Figure 2:**
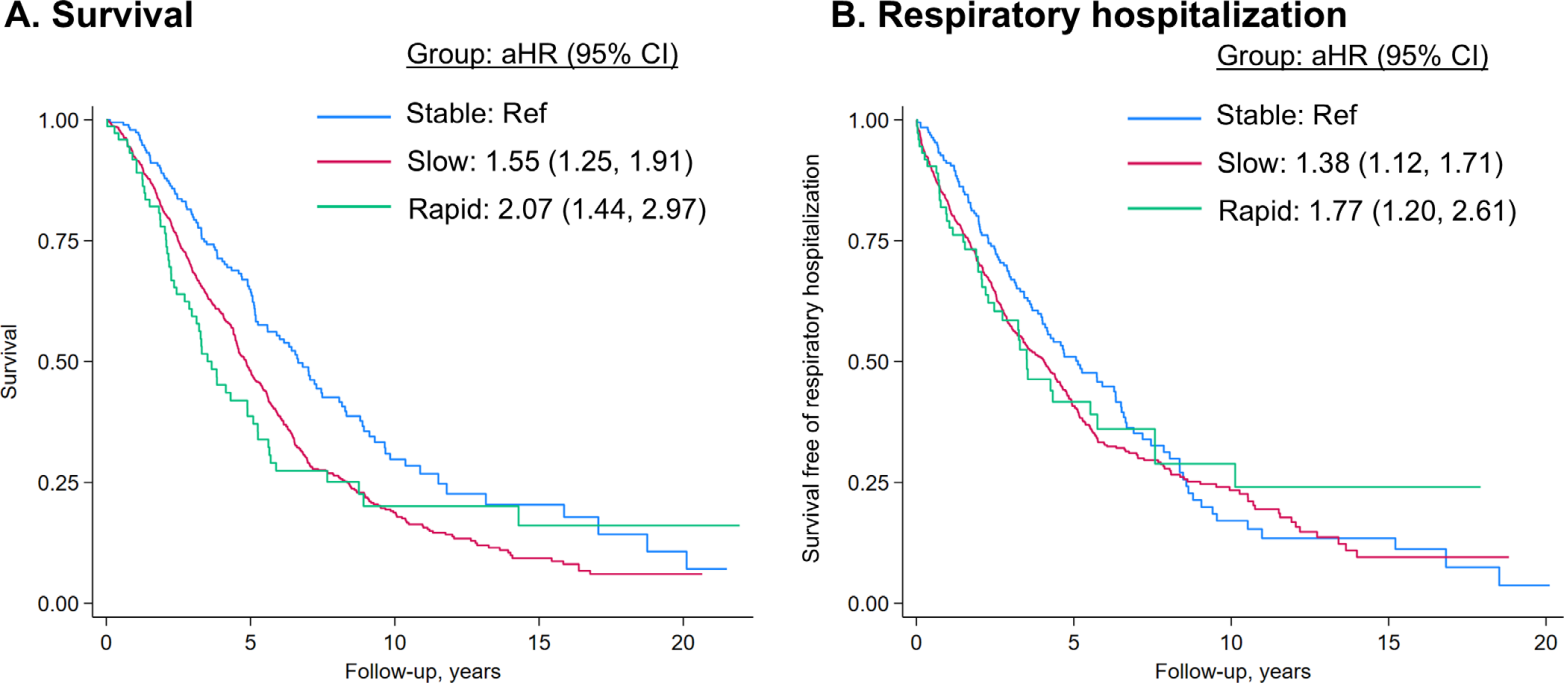
Kaplan–Meier curves of survival and respiratory hospitalization among patients with RA‐ILD by FVC trajectory group assignment in the primary RA‐ILD cohort. (A) Survival and (B) respiratory‐related hospitalizations throughout the study follow‐up by FVC trajectory group assignment. aHRs from multivariable models including age, sex, race, smoking status, calendar year period, comorbidity burden, and baseline FVC percent predicted. aHR, adjusted hazard ratio; CI, confidence interval; FVC, forced vital capacity; RA‐ILD, rheumatoid arthritis–associated interstitial lung disease.

Over 5,263 patient‐years of follow‐up, 727 deaths occurred. Compared to the stable group, the slow progression (adjusted hazard ratio [aHR] 1.55; confidence interval [CI] 1.25, 1.91) and rapid progression groups (aHR 2.07 [CI 1.44, 2.97]) were associated with poorer survival (Figure 2; Table 2).

**Table 2:** Survival and respiratory hospitalization risk by FVC trajectory group assignment in primary RA‐ILD cohort*

	Number of events / PY	IR (95% CI)^a^	aHR (95% CI)	*P* value
Mortality				
Overall	727 / 5,263	1.4 (1.3, 1.5)	–	–
Stable	113 / 1,113	1.0 (0.8, 1.2)	Ref.	–
Slow progression	561 / 3,814	1.5 (1.4, 1.6)	1.55 (1.25, 1.91)	<0.001
Rapid progression	53 / 337	1.6 (1.2, 2.1)	2.07 (1.44, 2.97)	<0.001
Respiratory hospitalization				
Overall	646 / 3,932	1.6 (1.5, 1.8)	–	–
Stable	117 / 832	1.4 (1.2, 1.7)	Ref.	–
Slow progression	488 / 2,842	1.7 (1.6, 1.9)	1.38 (1.12, 1.71)	0.002
Rapid progression	41 / 258	1.6 (1.2, 2.2)	1.77 (1.20, 2.61)	0.004

* Adjusted for age, sex, race, smoking status, diagnosis year period, number of baseline comorbidities, and baseline FVC percent predicted. aHR, adjusted hazard ratio; CI, confidence interval; FVC, forced vital capacity; IR, incidence rate; PY, person‐years; RA‐ILD, rheumatoid arthritis–associated interstitial lung disease.

^a^ Per 10 PY

#### Respiratory hospitalization

A total of 646 respiratory hospitalizations occurred over 3,932 patient‐years of follow‐up. The slow progression (aHR 1.38 [CI 1.12, 1.71]) and rapid progression (aHR 1.77 [CI 1.20, 2.61]) groups were associated with a higher respiratory hospitalization risk than the stable group (Figure 2; Table 2).

### Prognosis in overall RA‐ILD cohort by trajectories

#### Mortality

**Figure 3:**
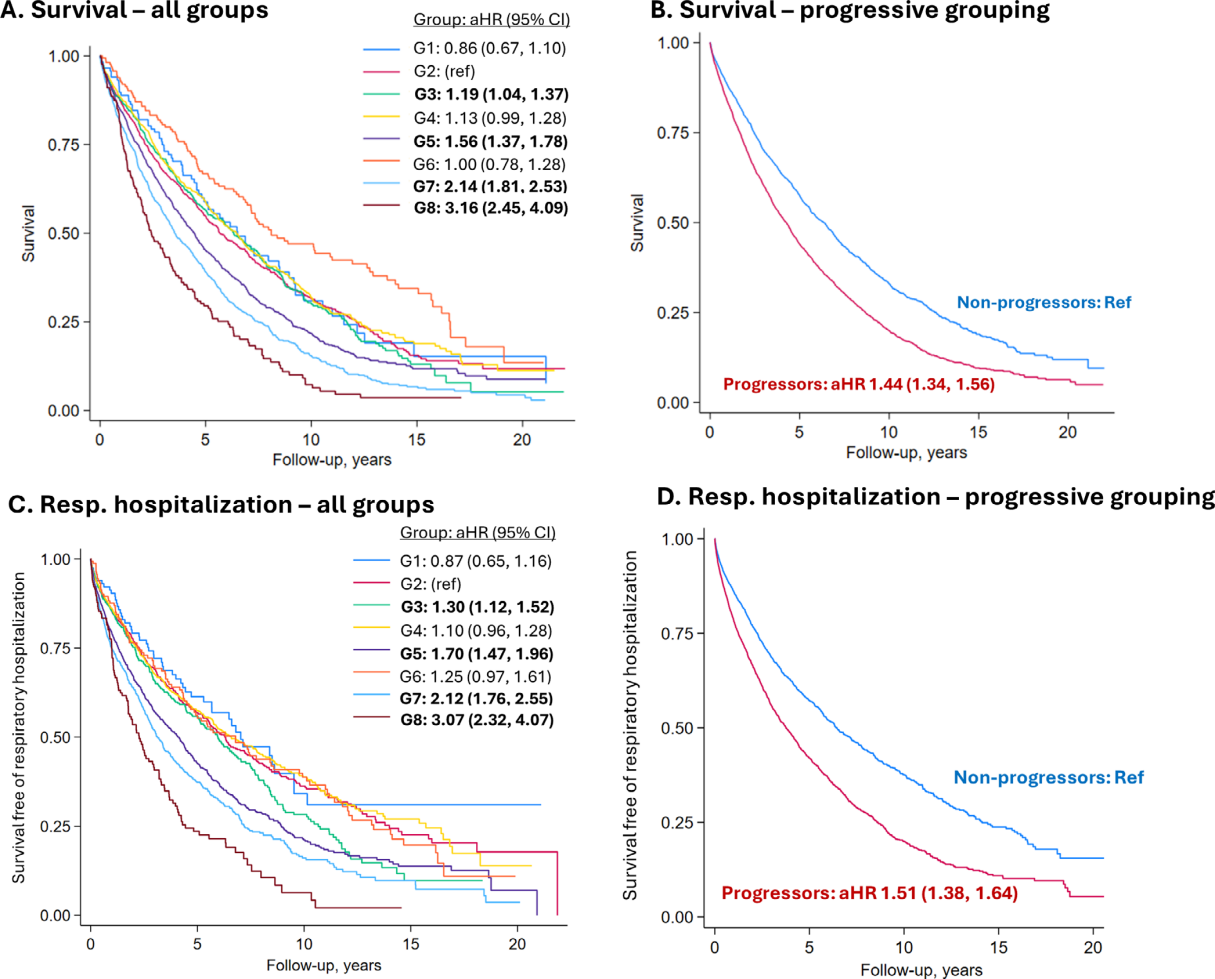
Kaplan–Meier curves of survival and respiratory hospitalization among patients with RA‐ILD by FVC trajectory group assignment in the overall RA‐ILD cohort. Survival and respiratory‐related hospitalization outcomes by FVC trajectory group assignment. (A) Depicts survival in all group assignments, whereas (B) depicts survival in progressive versus nonprogressive group assignments. (C) Depicts survival free of respiratory hospitalization in all groups, whereas (D) depicts survival free of respiratory hospitalization in progressive versus nonprogressive groups. aHRs from multivariable models including age, sex, race, smoking status, calendar year period, comorbidity burden, and baseline FVC percent predicted. aHR, adjusted hazard ratio; CI, confidence interval; FVC, forced vital capacity; G, group; RA‐ILD, rheumatoid arthritis–associated interstitial lung disease.

Over 24,780 patient‐years of follow‐up, 3,400 deaths occurred. Trajectory group 2 was used as the reference group given its size (20.5% of patients), normal initial FVC, and stable trajectory. All four progressive groups were individually associated with poorer survival in adjusted models (Figure 3A; Table 3). The aHR (95% CI) for these groups were 1.19 (1.04, 1.37) for group 3, 1.56 (1.37, 1.78) for group 4, 2.14 (1.81, 2.53) for group 7, and 3.16 (2.45, 4.09) for group 8. None of the nonprogressive trajectory groups were associated with higher mortality risk (aHR 0.89–1.07), including group 6, which had a reduced initial FVC value. When categorizing these eight groups into a binary of progressive versus nonprogressive, the progressive group was associated with a >1.4‐fold higher risk of death (aHR 1.44 [CI 1.34, 1.56]) independent of initial FVC (Figure 3B; Table 3).

**Table 3:** Survival and respiratory hospitalization risk by FVC trajectory group in overall RA‐ILD cohort*

	Number of events / PY	IR (95% CI)^a^	aHR (95% CI)	*P* value
Mortality				
Overall	3,400 / 24,780	1.4 (1.3, 1.4)	–	–
All groups				
Group 1	72 / 664	1.1 (0.9, 1.4)	0.86 (0.67, 1.10)	0.23
Group 2	614 / 5,094	1.2 (1.1, 1.3)	Ref.	–
Group 3^b^	299 / 2,488	1.2 (1.1, 1.3)	1.19 (1.04, 1.37)	0.01
Group 4	605 / 5,419	1.1 (1.0, 1.2)	1.13 (0.99, 1.28)	0.07
Group 5^b^	841 / 5,463	1.5 (1.4, 1.6)	1.56 (1.37, 1.78)	<0.001
Group 6	98 / 1,233	0.8 (0.7, 1.0)	1.00 (0.78, 1.28)	0.97
Group 7^b^	727 / 3,841	1.9 (1.8, 2.0)	2.14 (1.81, 2.53)	<0.001
Group 8^b^	144 / 577	2.5 (2.1, 2.9)	3.16 (2.45, 4.09)	<0.001
Progressive grouping				
Nonprogressive	1,389 / 12,410	1.1 (1.1, 1.2)	Ref.	–
Progressive	2,011 / 12,369	1.6 (1.6, 1.7)	1.44 (1.34, 1.56)	<0.001
Respiratory hospitalization				
Overall	2,763 / 19,404	1.4 (1.4, 1.5)	–	–
All groups				
Group 1	53 / 539	1.0 (0.8, 1.3)	0.87 (0.65, 1.16)	0.34
Group 2	473 / 4,204	1.1 (1.0, 1.2)	Ref.	–
Group 3^b^	252 / 1,912	1.3 (1.2, 1.5)	1.30 (1.12, 1.52)	0.001
Group 4	480 / 4,421	1.1 (1.0, 1.2)	1.10 (0.96, 1.28)	0.17
Group 5^b^	717 / 4,210	1.7 (1.6, 1.8)	1.70 (1.47, 1.96)	<0.001
Group 6	101 / 926	1.1 (0.9, 1.3)	1.25 (0.97, 1.61)	0.09
Group 7^b^	568 / 2,782	2.0 (1.9, 2.2)	2.12 (1.76, 2.55)	<0.001
Group 8^b^	119 / 410	2.9 (2.4, 3.5)	3.07 (2.32, 4.07)	<0.001
Progressive grouping				
Nonprogressive	1,107 / 10,090	1.1 (1.0, 1.2)	Ref.	–
Progressive	1,656 / 9,314	1.8 (1.7, 1.9)	1.51 (1.38, 1.64)	<0.001

* Adjusted for age, sex, race, smoking status, diagnosis year period, number of baseline comorbidities, and baseline FVC percent predicted. aHR, adjusted hazard ratio; CI, confidence interval; FVC, forced vital capacity; IR, incidence rate; PY, person‐years; RA‐ILD, rheumatoid arthritis–associated interstitial lung disease.

^a^ IR per 10 PY

^b^ Indicates a group categorized as progressive

#### Respiratory‐related hospitalization

Over 19,404 patient‐years of follow‐up, 2,763 patients with RA‐ILD experienced a respiratory‐related hospitalization. Referent to trajectory group 2, all progressive FVC trajectory groups had a higher risk of respiratory‐related hospitalization (Figure 3C; Table 3). The aHR (95% CI) for respiratory‐related hospitalization among these groups were 1.30 (1.12, 1.52) for group 3, 1.70 (1.47, 1.96) for group 5, 2.12 (1.76, 2.55) for group 7, and 3.07 (2.32, 4.07) for group 8. None of the groups categorized as nonprogressive were associated with respiratory hospitalization risk. Compared to the combined nonprogressive group, those with a progressive FVC trajectory had a significantly increased risk of respiratory‐related hospitalization (aHR 1.51 [CI 1.38, 1.64]; Figure 3D; Table 3).

## DISCUSSION

We aimed to evaluate the heterogeneity of RA‐ILD throughout its disease course by deriving unique clusters of patients with RA‐ILD based on longitudinal FVC trajectories. Using national VA data over a >20‐year period, we identified clusters of patients with RA‐ILD with stable, slowly progressive, and rapidly progressive disease courses. In total, 82.5% of patients in our primary RA‐ILD cohort (n = 1,092) and 54% of patients in our broader overall RA‐ILD cohort (n = 5,172) had a progressive disease phenotype. Progressive FVC decline trajectory clusters were strongly associated with both death and respiratory‐related hospitalization independent of initial FVC and other patient characteristics. Our findings further establish the heterogeneity of the RA‐ILD disease course and emphasize the need to develop approaches to prognosticate the disease course at, or shortly after, diagnosis. With such tools, clinicians will be better equipped to make treatment and monitoring decisions in RA‐ILD.

The findings from our national RA‐ILD study are in line with prior single center studies that identified three to four FVC trajectory groups in RA‐ILD.^12^, ^13^ One notable difference was that a higher proportion of patients with RA‐ILD in our cohort had a progressive disease course (82.5% vs 44% and 68%, respectively). In our broader overall RA‐ILD cohort, the number of trajectories identified was very similar to findings reported by Man et al in systemic sclerosis–associated ILD, who identified seven unique FVC trajectories.^23^ In both of our RA‐ILD cohorts, the rate of change was the parameter most closely associated with risk of death or hospitalization, with these progressive trajectories being associated with poor outcomes independent of initial FVC value. Findings from our overall RA‐ILD cohort demonstrate that the highest risk groups were patients with RA‐ILD that had the most rapidly progressive decline in FVC in addition to an impaired starting FVC value (eg, groups 7 and 8). Thus, reducing patients with RA‐ILD to a simple binary progressive and nonprogressive categorization may be an oversimplification of RA‐ILD progression. In contrast, a single nonprogressive RA‐ILD group appeared to summarize a nonprogressive disease course well, as incidence rates for death and respiratory‐related hospitalization outcomes were overlapping in the four nonprogressive groups in our overall RA‐ILD cohort. Our findings highlight the need for precise characterization of FVC change over time and the need for clinically feasible approaches to predict these unique disease trajectories.

There is no universally agreed upon definition for progression in RA‐ILD. Clinical practice guidelines for idiopathic pulmonary fibrosis (IPF) define progressive pulmonary fibrosis based on fulfilling two of three components: worsening respiratory symptoms, pulmonary physiology, and radiologic findings.^24^ The FVC component of pulmonary physiology is defined based on an absolute decrease of ≥5% predicted over a 1‐year follow‐up period. Clinical trials have used alternative definitions as part of their eligibility criteria and study outcomes. The INBUILD trial evaluating nintedanib in progressive pulmonary fibrosis required evidence of progression for study eligibility, using a definition of relative decline in FVC precent predicted by ≥10% or ≥5% plus worsening symptoms or imaging findings over the prior 24 months.^11^ The TRAIL1 trial evaluating pirfenidone in RA‐ILD selected a primary study outcome of progression based on decline in FVC percent by ≥10% or death.^25^ Compared to these prior definitions of progression, most of our progressive RA‐ILD groups had FVC change rates that were well below these thresholds, yet these groups still experienced a higher risk of death and respiratory‐related hospitalization during follow‐up. This suggests that reliance on the higher progression thresholds used as part of prior regulatory trials may be overly stringent to inform real‐world treatment decisions as patients with slow progression may experience improved disease‐related outcomes with treatment modifications.

Heterogeneity in RA‐ILD poses a major challenge to management. This study identified unique disease trajectories based on FVC values alone, but FVC variability represents only one contributor to RA‐ILD heterogeneity alongside ILD pattern and genetic predisposition, among others. Recognizing the substantial heterogeneity in RA‐ILD, precise disease subsetting has the potential to guide management decisions. However, what factors are most useful for separating these subsets is not known. In this study, we found standard demographic and clinical factors were only modestly associated with different FVC trajectories. This suggests variability in FVC trajectory is more likely related to heterogeneity in other disease‐related factors and highlights the need for more advanced phenotyping, such as broader autoantibody profiling or proteomic and radiomic evaluation, of RA‐ILD to identify factors underlying these unique disease trajectories. Supporting this, translational studies have identified unique peripheral blood biomarker and proteomic signatures associated with the presence of RA‐ILD.^26^, ^27^ A small proof of concept study evaluated whole lung radiomic analysis in 30 patients with RA‐ILD and found that a subset of the 22 unique radiomic features extracted predicted overall survival.^28^ Further, Humphries and colleagues used data‐driven texture analysis for chest CT quantification of fibrosis in two independent RA‐ILD cohorts to predict RA‐ILD survival.^29^ The discovery and validation of such approaches and their application to clinical care early in the RA‐ILD course could prove to be highly informative for forecasting disease trajectories and fostering earlier intervention, rather than the current status quo of waiting for disease progression to occur.

Limitations to the study include the potential lack of generalizability given the male predominance of the cohort. The high frequency of comorbid COPD likely reflects the high cigarette smoking rates and may also impact generalizability. Our cohort consisted of both prevalent and incident RA‐ILD cases, which may have deemphasized FVC trajectories during the early disease period. FVC values were obtained through real‐world clinical care, which led to variable testing frequency and testing intervals. Although we used validated algorithms and NLP tools,^18^, ^19^ there may be misclassification of RA‐ILD status and FVC values. ILD pattern was not available in our data. Future studies are needed to understand how RA‐ILD FVC trajectories may differ based on ILD pattern. Our approach for trajectory modeling grouped patients with RA‐ILD by the entirety of their FVC data into single trajectory over time. With the current study design and time period evaluated, we were not positioned to evaluate how treatment modifications (eg, DMARDs and/or antifibrotics), RA disease flares, ILD flares, or environmental exposures occurring during the disease course may have influenced FVC trajectory. FVC values and the clinical events of death and respiratory hospitalization were assessed over the same follow‐up period, which could result in immortal time bias, though our findings of FVC decline associating with mortality and respiratory hospitalization risk are consistent with other studies.^30^

In conclusion, we identified unique clusters of patients with RA‐ILD based on FVC trajectories using large, real‐world data over a >20‐year period. These clusters were defined by variability in the rate of decline throughout the follow‐up period as well as initial FVC values. FVC trajectory clusters that reflected progression were strongly associated with both survival and respiratory‐related hospitalization risk, despite most progressive groups not reaching thresholds for progression used in IPF clinical practice guidelines or clinical trials. These findings illustrate the heterogeneity of the RA‐ILD disease course and highlight the poor prognosis even for those who experience “slow” ILD progression. Future studies are needed to develop methods that can better distinguish unique disease subsets early in the disease course to inform proactive management decisions.

## AUTHOR CONTRIBUTIONS

All authors contributed to at least one of the following manuscript preparation roles: conceptualization AND/OR methodology, software, investigation, formal analysis, data curation, visualization, and validation AND drafting or reviewing/editing the final draft. As corresponding author, Dr England confirms that all authors have provided the final approval of the version to be published, and takes responsibility for the affirmations regarding article submission (eg, not under consideration by another journal), the integrity of the data presented, and the statements regarding compliance with institutional review board/Declaration of Helsinki requirements.

## ROLE OF THE STUDY SPONSOR

Boehringer‐Ingelheim had no role in the study design or in the collection, analysis, or interpretation of the data, the writing of the manuscript, or the decision to submit the manuscript for publication. Publication of this article was not contingent upon approval by Boehringer‐Ingelheim.

## Supporting information


**Disclosure Form**:

**Data S1** Supporting Information
