# Supplementary material for: Heterogeneity of Rheumatoid Arthritis–Associated Interstitial Lung Disease by Longitudinal Forced Vital Capacity Trajectory and Associations With Disease Outcomes
Source: Arthritis Care Res (Hoboken). 2025 Nov 13;78(2):249–58. doi: 10.1002/acr.25620 (PMC12919699; doi:10.1002/acr.25620)
Supplement: Supplementary file 2 — Data S1 Supporting Information [file ACR-78-249-s002.docx]

**SUPPLEMENTARY MATERIALS**

Heterogeneity of Rheumatoid Arthritis-Associated Interstitial Lung Disease by Longitudinal Forced Vital Capacity Trajectory and Associations with Disease Outcomes

England BR et al.

**Supplemental Table 1**. Performance metrics for selection of FVC trajectory groups in primary RA-ILD cohort.

**Supplemental Table 2**. Patient characteristics by FVC trajectory group assignment in primary RA-ILD cohort.

**Supplemental Table 3**. Performance metrics for selection of FVC trajectory groups in overall RA-ILD cohort.

**Supplemental Table 4**. Patient characteristics by progressive vs. non-progressive FVC trajectory group assignment in overall RA-ILD cohort.

**Supplemental Table 5**. Patient characteristics by FVC trajectory group assignment in overall RA-ILD cohort.

**Supplemental Table 1.** Performance metrics for selection of FVC trajectory groups in primary RA-ILD cohort.

| Number of trajectory groups | BIC | AIC | BIC diff | AIC diff | Smallest group size (%) |
| --- | --- | --- | --- | --- | --- |
| 1 | -16297.67 | -16290.17 |  |  |  |
| 2 | -15926.28 | -15911.29 | -371.39 | -378.88 | 37.5 |
| 3 | -15678.84 | -15656.36 | -247.44 | -254.93 | 6.7 |
| 4 | -15517.09 | -15487.12 | -161.75 | -169.24 | 1.0 |
| 5 | -15393.00 | -15355.53 | -124.09 | -131.59 | 0.3 |
| 6 | -15403.49 | -15358.53 | 10.49 | 3.00 | 0.3 |
| 7 | -15349.29 | -15296.83 | -54.20 | -61.70 | 0.3 |
| 8 | -15322.71 | -15262.76 | -26.58 | -34.07 | 0.3 |

AIC and BIC differences are compared to above row

Optimal group number based on AIC, BIC, and group size highlighted in green.

Abbreviations: AIC, Akaiki Information Criteria; BIC, Bayesian Information Criteria; diff, difference

**Supplemental Table 2.** Patient characteristics by FVC trajectory group in primary cohort.

|  | **Overall**  (n=1,092) | **Stable**  (n=191) | **Slow progression**  (n=828) | **Rapid progression**  (n=73) |  |
| --- | --- | --- | --- | --- | --- |
| Age, years | 68.0 (9.5) | 66.2 (9.4) | 68.3 (9.4) | 68.8 (9.5) |  |
| Male | 1,012 (92.7%) | 173 (90.6%) | 771 (93.1%) | 68 (93.2%) |  |
| Race |  |  |  |  |  |
| Asian/multiple races | 24 (2.2%) | 1 (0.5%) | 23 (2.8%) | 0 (0.0%) |  |
| Black | 162 (14.8%) | 26 (13.6%) | 126 (15.2%) | 10 (13.7%) |  |
| White | 782 (71.6%) | 149 (78.0%) | 585 (70.7%) | 48 (65.8%) |  |
| Missing | 124 (11.4%) | 15 (7.9%) | 94 (11.4%) | 15 (20.5%) |  |
| Calendar year |  |  |  |  |  |
| 1999-2005 | 284 (26.0%) | 50 (26.2%) | 210 (25.4%) | 24 (32.9%) |  |
| 2006-2011 | 256 (23.4%) | 50 (26.2%) | 186 (22.5%) | 20 (27.4%) |  |
| 2012-2021 | 552 (50.5%) | 91 (47.6%) | 432 (52.2%) | 29 (39.7%) |  |
| Smoking status |  |  |  |  |  |
| Never | 133 (12.2%) | 29 (15.2%) | 100 (12.1%) | 4 (5.5%) |  |
| Former | 346 (31.7%) | 53 (27.7%) | 267 (32.2%) | 26 (35.6%) |  |
| Current | 463 (42.4%) | 84 (44.0%) | 351 (42.4%) | 28 (38.4%) |  |
| Missing | 150 (13.7%) | 25 (13.1%) | 110 (13.3%) | 15 (20.5%) |  |
| Comorbidity burden | 10.1 (4.8) | 10.9 (5.0) | 10.1 (4.7) | 8.8 (4.4) |  |
| COPD | 481 (44.0%) | 103 (53.9%) | 345 (41.7%) | 33 (45.2%) |  |
| RF/CCP antibody status |  |  |  |  |  |
| Seronegative | 190 (17.4%) | 38 (19.9%) | 138 (16.7%) | 14 (19.2%) |  |
| Seropositive | 747 (68.4%) | 133 (69.6%) | 568 (68.6%) | 46 (63.0%) |  |
| Missing | 155 (14.2%) | 20 (10.5%) | 122 (14.7%) | 13 (17.8%) |  |
| ESR/CRP |  |  |  |  |  |
| Normal | 230 (21.1%) | 40 (20.9%) | 176 (21.3%) | 14 (19.2%) |  |
| High | 696 (63.7%) | 123 (64.4%) | 527 (63.6%) | 46 (63.0%) |  |
| Missing | 166 (15.2%) | 28 (14.7%) | 125 (15.1%) | 13 (17.8%) |  |
| Prednisone | 740 (67.8%) | 141 (73.8%) | 550 (66.4%) | 49 (67.1%) |  |
| csDMARDs | 708 (64.8%) | 116 (60.7%) | 547 (66.1%) | 45 (61.6%) |  |
| b/tsDMARDs | 313 (28.7%) | 49 (25.7%) | 246 (29.7%) | 18 (24.7%) |  |
| Antifibrotic use | 2 (0.2%) | 0 (0.0%) | 2 (0.2%) | 0 (0.0%) |  |

Values mean (SD) or n (%)

Abbreviations: b/tsDMARD, biologic/targeted-synthetic disease-modifying anti-rheumatic drug; CCP, cyclic-citrullinated peptide; COPD, chronic obstructive pulmonary disease; CRP, C-reactive protein; csDMARD, conventional-synthetic disease-modifying anti-rheumatic drug; ESR, erythrocyte sedimentation rate; RF, rheumatoid factor

**Supplemental Table 3.** Performance metrics for selection of FVC trajectory groups in overall RA-ILD cohort.

| Number of trajectory groups | BIC | AIC | BIC diff | AIC diff | Smallest group size (%) |
| --- | --- | --- | --- | --- | --- |
| 1 | -78394.59 | -78384.77 |  |  |  |
| 2 | -75254.34 | -75234.69 | -3140.25 | -3150.08 | 45.3 |
| 3 | -74190.51 | -74161.03 | -1063.83 | -1073.66 | 21.9 |
| 4 | -73739.13 | -73699.83 | -451.38 | -461.20 | 12.4 |
| 5 | -73570.16 | -73521.03 | -168.97 | -178.80 | 4.3 |
| 6 | -73475.34 | -73398.38 | -94.82 | -122.65 | 2.5 |
| 7 | -73400.81 | -73332.03 | -74.53 | -66.35 | 3.4 |
| 8 | -73337.86 | -73259.25 | -62.95 | -72.78 | 2.3 |
| 9 | -73301.64 | -73213.21 | -36.22 | -46.04 | 1.2 |
| 10 | -73283.57 | -73185.31 | -18.07 | -27.90 | 2.1 |
| 11 | -73263.87 | -73155.78 | -19.70 | -29.53 | 1.0 |
| 12 | -73249.91 | -73132.00 | -13.96 | -23.78 | 0.5 |
| 13 | -73234.56 | -73106.81 | -15.35 | -25.19 | 0.5 |
| 14 | -73228.26 | -73090.69 | -6.30 | -16.12 | 0.5 |
| 15 | -73207.33 | -73059.93 | -20.93 | -30.76 | 0.5 |

AIC and BIC differences are compared to above row

Optimal group number based on AIC, BIC, and group size highlighted in green.

Abbreviations: AIC, Akaiki Information Criteria; BIC, Bayesian Information Criteria; diff, difference

**Supplemental Table 4**. Patient characteristics by progressive vs. non-progressive FVC trajectory assignment in overall RA-ILD cohort.

|  | **Overall**  (n=5,172) | **Progressive** (n=2,793) | **Non-progressive** (n=2,379) |
| --- | --- | --- | --- |
| Age, years | 68.8 (9.9) | 68.2 (9.7) | 69.4 (10.0) |
| Male | 4,776 (92.3%) | 2,590 (92.7%) | 2,186 (91.9%) |
| Race |  |  |  |
| Asian/multiple races | 122 (2.4%) | 68 (2.4%) | 54 (2.3%) |
| Black | 708 (13.7%) | 377 (13.5%) | 331 (13.9%) |
| White | 3,882 (75.1%) | 2,084 (74.6%) | 1,798 (75.6%) |
| Missing | 460 (8.9%) | 264 (9.5%) | 196 (8.2%) |
| Calendar year |  |  |  |
| 1999-2005 | 943 (18.2%) | 581 (20.8%) | 362 (15.2%) |
| 2006-2011 | 1,382 (26.7%) | 767 (27.5%) | 615 (25.9%) |
| 2012-2021 | 2,847 (55.0%) | 1,445 (51.7%) | 1,402 (58.9%) |
| Smoking status |  |  |  |
| Never | 606 (11.7%) | 336 (12.0%) | 270 (11.3%) |
| Former | 1,619 (31.3%) | 899 (32.2%) | 720 (30.3%) |
| Current | 2,331 (45.1%) | 1,177 (42.1%) | 1,154 (48.5%) |
| Missing | 616 (11.9%) | 381 (13.6%) | 235 (9.9%) |
| Comorbidity burden | 11.4 (5.3) | 11.4 (5.4) | 11.3 (5.2) |
| COPD | 2,569 (49.7%) | 1,434 (51.3%) | 1,135 (47.7%) |
| RF/CCP antibody status |  |  |  |
| Seronegative | 903 (17.5%) | 472 (16.9%) | 431 (18.1%) |
| Seropositive | 3,479 (67.3%) | 1,857 (66.5%) | 1,622 (68.2%) |
| Missing | 790 (15.3%) | 464 (16.6%) | 326 (13.7%) |
| ESR/CRP |  |  |  |
| Normal | 1,143 (22.1%) | 565 (20.2%) | 578 (24.3%) |
| High | 3,402 (65.8%) | 1,854 (66.4%) | 1,548 (65.1%) |
| Missing | 627 (12.1%) | 374 (13.4%) | 253 (10.6%) |
| Prednisone | 3,744 (72.4%) | 2,025 (72.5%) | 1,719 (72.3%) |
| csDMARDs | 3,573 (69.1%) | 1,877 (67.2%) | 1,696 (71.3%) |
| b/tsDMARDs | 1,702 (32.9%) | 869 (31.1%) | 833 (35.0%) |
| Antifibrotic use | 9 (0.2%) | 6 (0.2%) | 3 (0.1%) |

Values mean (SD) or n (%)

Abbreviations: b/tsDMARD, biologic/targeted-synthetic disease-modifying anti-rheumatic drug; CCP, cyclic-citrullinated peptide; COPD, chronic obstructive pulmonary disease; CRP, C-reactive protein; csDMARD, conventional-synthetic disease-modifying anti-rheumatic drug; ESR, erythrocyte sedimentation rate; RF, rheumatoid factor

**Supplemental Table 5**. Patient characteristics by FVC trajectory assignment in overall RA-ILD cohort.

|  | Overall | Group 1 | Group 2 | **Group 3** | Group 4 | **Group 5** | Group 6 | **Group 7** | **Group 8** |
| --- | --- | --- | --- | --- | --- | --- | --- | --- | --- |
|  | (n=5,172) | (n=117) | (n=1,062) | **(n=450)** | (n=1,035) | **(n=1,237)** | (n=165) | **(n=938)** | **(n=168)** |
| Age, years | 68.8 (9.9) | 70.3 (10.7) | 70.6 (9.6) | **68.1 (9.7)** | 68.6 (10.0) | **68.5 (9.5)** | 66.2 (11.1) | **68.2 (9.9)** | **66.5 (10.1)** |
| Male | 4,776 (92.3%) | 110 (94.0%) | 992 (93.4%) | **420 (93.3%)** | 939 (90.7%) | **1,146 (92.6%)** | 145 (87.9%) | **874 (93.2%)** | **150 (89.3%)** |
| Race |  |  |  |  |  |  |  |  |  |
| White | 3,882 (75.1%) | 84 (71.8%) | 802 (75.5%) | **341 (75.8%)** | 789 (76.2%) | **921 (74.5%)** | 123 (74.5%) | **697 (74.3%)** | **125 (74.4%)** |
| Black | 708 (13.7%) | 16 (13.7%) | 145 (13.7%) | **66 (14.7%)** | 144 (13.9%) | **171 (13.8%)** | 26 (15.8%) | **120 (12.8%)** | **20 (11.9%)** |
| Asian/multiple | 122 (2.4%) | 2 (1.7%) | 23 (2.2%) | **11 (2.4%)** | 21 (2.0%) | **31 (2.5%)** | 8 (4.8%) | **23 (2.5%)** | **3 (1.8%)** |
| Missing | 460 (8.9%) | 15 (12.8%) | 92 (8.7%) | **32 (7.1%)** | 81 (7.8%) | **114 (9.2%)** | 8 (4.8%) | **98 (10.4%)** | **20 (11.9%)** |
| Calendar year |  |  |  |  |  |  |  |  |  |
| 1999-2005 | 943 (18.2%) | 15 (12.8%) | 168 (15.8%) | **73 (16.2%)** | 150 (14.5%) | **235 (19.0%)** | 29 (17.6%) | **223 (23.8%)** | **50 (29.8%)** |
| 2006-2011 | 1,382 (26.7%) | 35 (29.9%) | 245 (23.1%) | **133 (29.6%)** | 279 (27.0%) | **331 (26.8%)** | 56 (33.9%) | **251 (26.8%)** | **52 (31.0%)** |
| 2012-2021 | 2,847 (55.0%) | 67 (57.3%) | 649 (61.1%) | **244 (54.2%)** | 606 (58.6%) | **671 (54.2%)** | 80 (48.5%) | **464 (49.5%)** | **66 (39.3%)** |
| Smoking status |  |  |  |  |  |  |  |  |  |
| Never | 606 (11.7%) | 9 (7.7%) | 107 (10.1%) | **39 (8.7%)** | 130 (12.6%) | **135 (10.9%)** | 24 (14.5%) | **141 (15.0%)** | **21 (12.5%)** |
| Former | 1,619 (31.3%) | 31 (26.5%) | 339 (31.9%) | **147 (32.7%)** | 303 (29.3%) | **400 (32.3%)** | 47 (28.5%) | **295 (31.4%)** | **57 (33.9%)** |
| Current | 2,331 (45.1%) | 68 (58.1%) | 515 (48.5%) | **218 (48.4%)** | 503 (48.6%) | **552 (44.6%)** | 68 (41.2%) | **350 (37.3%)** | **57 (33.9%)** |
| Missing | 616 (11.9%) | 9 (7.7%) | 101 (9.5%) | **46 (10.2%)** | 99 (9.6%) | **150 (12.1%)** | 26 (15.8%) | **152 (16.2%)** | **33 (19.6%)** |
| Comorbidity burden | 11.4 (5.3) | 10.5 (4.2) | 11.1 (5.3) | **11.3 (5.2)** | 11.3 (5.2) | **11.5 (5.3)** | 13.6 (5.5) | **11.4 (5.6)** | **11.6 (6.0)** |
| COPD | 2,569 (49.7%) | 73 (62.4%) | 467 (44.0%) | **223 (49.6%)** | 482 (46.6%) | **605 (48.9%)** | 113 (68.5%) | **509 (54.3%)** | **97 (57.7%)** |
| RF/CCP |  |  |  |  |  |  |  |  |  |
| Seronegative | 903 (17.5%) | 28 (23.9%) | 173 (16.3%) | **56 (12.4%)** | 193 (18.6%) | **201 (16.2%)** | 37 (22.4%) | **173 (18.4%)** | **42 (25.0%)** |
| Seropositive | 3,479 (67.3%) | 70 (59.8%) | 742 (69.9%) | **339 (75.3%)** | 702 (67.8%) | **849 (68.6%)** | 108 (65.5%) | **580 (61.8%)** | **89 (53.0%)** |
| Missing | 790 (15.3%) | 19 (16.2%) | 147 (13.8%) | **55 (12.2%)** | 140 (13.5%) | **187 (15.1%)** | 20 (12.1%) | **185 (19.7%)** | **37 (22.0%)** |
| ESR/CRP |  |  |  |  |  |  |  |  |  |
| Normal | 1,143 (22.1%) | 32 (27.4%) | 250 (23.5%) | **95 (21.1%)** | 250 (24.2%) | **258 (20.9%)** | 46 (27.9%) | **183 (19.5%)** | **29 (17.3%)** |
| High | 3,402 (65.8%) | 70 (59.8%) | 701 (66.0%) | **295 (65.6%)** | 673 (65.0%) | **820 (66.3%)** | 104 (63.0%) | **624 (66.5%)** | **115 (68.5%)** |
| Missing | 627 (12.1%) | 15 (12.8%) | 111 (10.5%) | **60 (13.3%)** | 112 (10.8%) | **159 (12.9%)** | 15 (9.1%) | **131 (14.0%)** | **24 (14.3%)** |
| Prednisone | 3,744 (72.4%) | 80 (68.4%) | 755 (71.1%) | **327 (72.7%)** | 755 (72.9%) | **890 (71.9%)** | 129 (78.2%) | **691 (73.7%)** | **117 (69.6%)** |
| csDMARDs | 3,573 (69.1%) | 78 (66.7%) | 775 (73.0%) | **314 (69.8%)** | 733 (70.8%) | **844 (68.2%)** | 110 (66.7%) | **628 (67.0%)** | **91 (54.2%)** |
| b/tsDMARDs | 1,702 (32.9%) | 44 (37.6%) | 388 (36.5%) | **151 (33.6%)** | 349 (33.7%) | **409 (33.1%)** | 52 (31.5%) | **272 (29.0%)** | **37 (22.0%)** |
| Antifibrotics | 9 (0.2%) | 0 (0.0%) | 2 (0.2%) | **0 (0.0%)** | 1 (0.1%) | **2 (0.2%)** | 0 (0.0%) | **2 (0.2%)** | **2 (1.2%)** |

Values mean (SD) or n (%). Bolded groups (3, 5, 7, 8) are those categorized as progressive.

Abbreviations: b/tsDMARD, biologic/targeted-synthetic disease-modifying anti-rheumatic drug; CCP, cyclic-citrullinated peptide; COPD, chronic obstructive pulmonary disease; CRP, C-reactive protein; csDMARD, conventional-synthetic disease-modifying anti-rheumatic drug; ESR, erythrocyte sedimentation rate; RF, rheumatoid factor
